# Editorial: Translational research in the diagnosis and development of therapeutics for peritoneal surface malignancies

**DOI:** 10.3389/fonc.2023.1232993

**Published:** 2023-07-10

**Authors:** Claramae Shulyn Chia, Yan Li, Wim Ceelen, Chin-Ann Johnny Ong

**Affiliations:** ^1^ Department of Sarcoma, Peritoneal and Rare Tumours (SPRinT), Division of Surgery and Surgical Oncology, National Cancer Centre Singapore, Singapore, Singapore; ^2^ Department of Sarcoma, Peritoneal and Rare Tumours (SPRinT), Division of Surgery and Surgical Oncology, Singapore General Hospital, Singapore, Singapore; ^3^ SingHealth Duke-NUS Oncology Academic Clinical Program, Duke-NUS Medical School, Singapore, Singapore; ^4^ SingHealth Duke-NUS Surgery Academic Clinical Program, Duke-NUS Medical School, Singapore, Singapore; ^5^ Department of Peritoneal Cancer Surgery and Pathology, Beijing Tsinghua Changgung Hospital, Beijing, China; ^6^ Department of Gastrointestinal Surgery, Ghent University Hospital, Ghent, Belgium; ^7^ Laboratory of Applied Human Genetics, Division of Medical Sciences, National Cancer Centre Singapore, Singapore, Singapore; ^8^ Institute of Molecular and Cell Biology, ASTAR Research Entities, Singapore, Singapore

**Keywords:** translational, therapeutics, peritoneal surface malignancies, diagnosis, research

Peritoneal surface malignancies (PSM) refer to a heterogenous group of primary and metastatic cancers that can arise from intraperitoneal (e.g. gastrointestinal, gynecological) or extraperitoneal (e.g. lung, breast) organs (1). The structure of healthcare systems in most countries are organ-centric, leading to a phenomenon where PSM are often managed as an end-stage disease by each subspeciality. Accurate diagnosis of PSM and complete surgical extirpation are fraught with challenges (2). Coupled with a duet of biologically advanced disease and suboptimal knowledge and expertise in dealing with this disease, prognosis of patients with PSM is uniformly poor in many countries.

Fortunately, with the recognition of PSM as a treatable phenomenon that transcends multiple intraperitoneal and extraperitoneal organs, two major organisations (International Society for the Study of Pleura and Peritoneum (ISSPP) and The Peritoneal Surface Oncology Group International (PSOGI)) were set up to amalgamate the surgical and medical expertise to combat this disease collectively (3, 4). Clinical guidelines are continually being updated and management algorithms constantly debated to reach clinical consensus by the above mentioned consortia. However, the future of PSM management as in all other diseases lies in devising novel diagnostic and therapeutic strategies. Progressing translational research in this arena hence remains the cornerstone to improve patient outcome. It is with this vision that we started this Research Topic, with the aim to highlight areas of deficiencies and continual scientific research that can feed into clinical algorithms for PSM management.

Helderman et al. performed a pragmatic study to examine the effects of hyperthermia and the duration of intra-peritoneal chemotherapy *in vivo*. The authors demonstrated that the efficacy of intraperitoneal chemotherapy (oxaliplatin and mitomycin C) in *in vitro* and *in vivo* models is dependent on the temperature of the perfusate and treatment duration. Hyperthermic chemotherapy applied at 41-42° C for 90 minutes improved drug uptake, induced apoptosis and decreased proliferation compared to lower temperatures and shorter duration without increasing the toxicity in normal tissue. With the failure of the PRODIGE 7 study (5) to demonstrate superiority of the hyperthermic chemotherapy in improving patient outcome compared to control, we eagerly await the results of the GECOP-MMC trial (Clinicaltrials.gov identifier: NCT05250648), which studies the effect of hyperthermic mitomycin C in reducing tumour recurrence after cytoreductive surgery and hyperthermic intra-peritoneal chemotherapy (HIPEC) (6). A positive result from this randomised controlled study will be testament to the importance of translational research to inform appropriate trial design.

2 other studies by Valenzuela-Molina et al. and Löke et al. explore the biology of PSM and attempt to combine treatment planning software with an atomically accurate 3D-printed phantom of a female peritoneum. The elegant study by Valenzuela-Molina et al. demonstrated low intratumoral oxygen levels in psudomyxoma peritonei samples with consequent HIF-1α levels. This could pave the way for potential novel therapeutic strategies in regulation of the hypoxia pathway in PSM. Löke et al., on the other hand, combined software simulations with experimental validation to define the use of a thermal module to account for forced convection during HIPEC. Several experimental conditions including catheter setups, inflow temperatures and flow rates were considered and compared to simulations to determine the accuracy of the treatment planning software. This study demonstrated the potential of harnessing software simulation to guide and evaluate treatment strategies by optimising HIPEC treatments.

This series would not be complete without defining the effect of the genomic landscape of PSM on patient outcome. Nguyen et al. explored the tumour molecular signatures of peritoneal metastases across multiple histological subtypes and correlated this with progression-free survival of patients. While the sample size is limited, the authors identified *AGAP5* as a potential prognostic gene among other putative driver genes identified. This study complements multiple other genome wide studies, which define the effect of the genomic, transcriptomic and epigenomic landscape on patient outcome and the development of novel therapeutics (7, 8).

This short series of articles in this Research Topic highlights multiple arenas of future translational research to improve PSM management. Conceptually, the areas of research needed are summarised in **Figure 1**. Beyond the areas covered in these series, fundamental research into the tumour microenvironment that inform diagnostics, detection and therapeutics are also critical milestones for the advancement of care for PSM patients (9–13).

**Figure 1:**
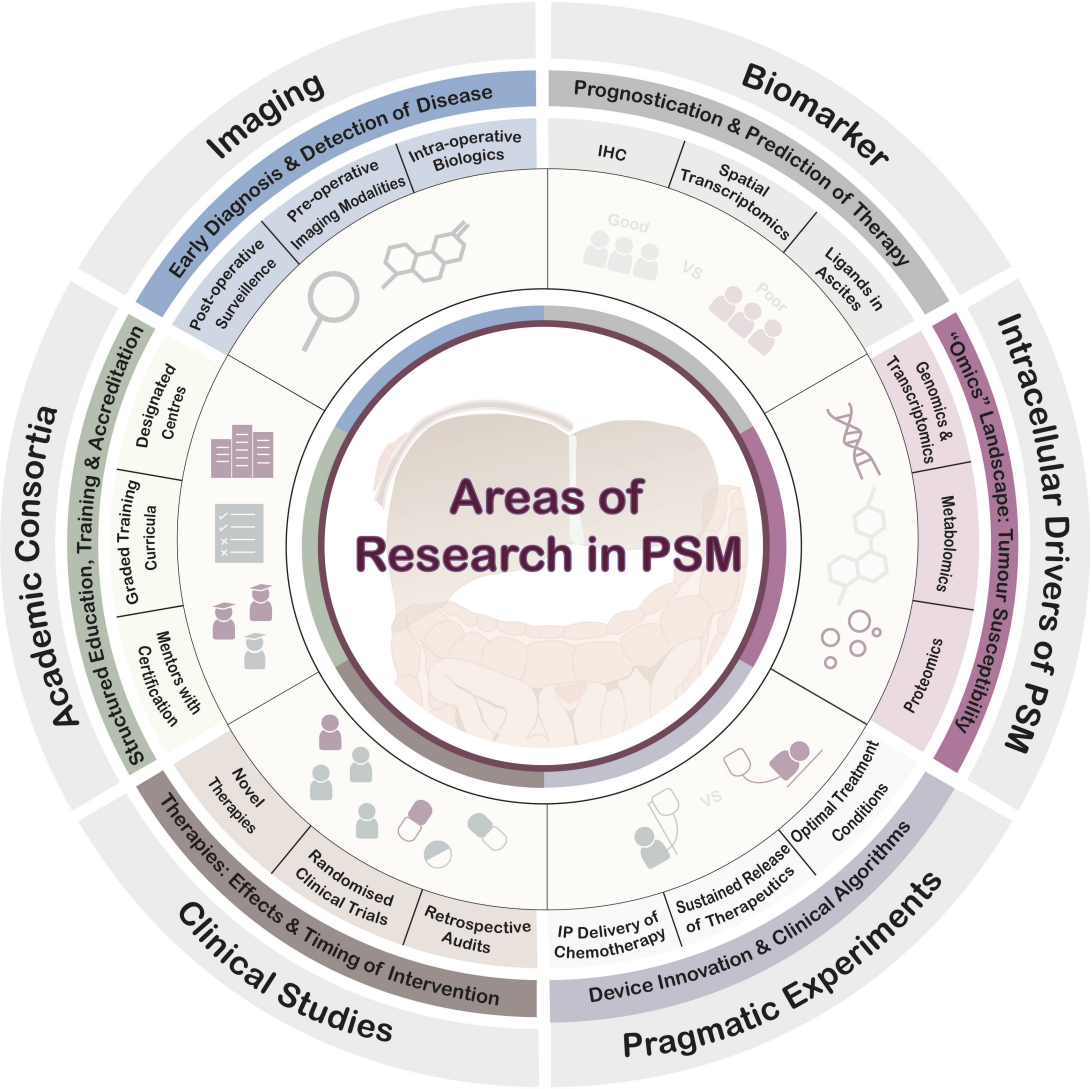
Key areas of research in PSM.

Finally, it is only with the tripartite development of clinical guidelines championed by consortia (e.g. ISSPP, PSOGI), translational research to move the needle for novel interventions, and rigorous testing via clinical studies, that care for PSM could improve substantially in the near future.

## Author contributions

CSC, YL, WC and C-AJO wrote, reviewed and approved the manuscript for submission.
